# The mediating role of psychological capital on the relationship between authentic leadership and nurses’ caring behavior: a cross-sectional study

**DOI:** 10.1186/s12912-023-01610-4

**Published:** 2023-11-22

**Authors:** Guowen Zhang, Wen Tian, Ying Zhang, Juanjuan Chen, Xiaohong Zhang, Wenfeng Lin, Huiping Li, Liqin Sun, Baozhen Cheng, Hui Ding, Guiqi Song

**Affiliations:** 1 Department of Nursing, Department of Emergency, Division of Life Sciences and Medicine, The First Affiliated Hospital of USTC, University of Science and Technology of China, Hefei, 230001 China; 2 School of Nursing, Anhui Medical University, Hefei, 230602 China; 3 The First Affiliated Hospital of Anhui University of Chinese Medicine, Hefei, 230031 China; 4 Department of Outpatient, Division of Life Sciences and Medicine, The First Affiliated Hospital of USTC, University of Science and Technology of China, Hefei, 230001 China; 5 Anhui Mental Health Center, Hefei, 230022 China; 6 Department of Education, Division of Life Sciences and Medicine, The First Affiliated Hospital of USTC, University of Science and Technology of China, Hefei, 230001 China

**Keywords:** Caring, Leadership, Mediation analysis, Nurses

## Abstract

**Background:**

Caring behavior among nurses would have an impact on patient outcomes. External organizational job resources and personal internal psychological resources are correlated to nurses’ caring behavior. Authentic leadership and psychological capital were shown to be correlated with nurses’ caring behavior in previous studies. However, the relationships among the three are nevertheless unclear. This study aimed to examine if psychological capital could act as a mediator between nursing managers’ authentic leadership and nurses’ caring behavior.

**Methods:**

In December 2021, a total of 3,662 nurses were recruited from 37 hospitals in Anhui Province, China. They filled out online surveys, including general demographic information, the Authentic Leadership Questionnaire, the Psychological Capital Questionnaire, and the Caring Behavior Inventory. Structural Equation Modeling and the bootstrapping procedure were used to examine the mediating role of psychological capital.

**Results:**

The scores of authentic leadership, psychological capital, and caring behavior of 3,495 nurses were 52.04 ± 13.24, 96.89 ± 17.78, and 104.28 ± 17.01, respectively. Psychological capital significantly mediated the relationship between authentic leadership and nurses’ caring behavior (*β* = 0.378, *p* < 0.001, 95% confidence interval: 0.350 ~ 0.402), which made up 78.75% of the total impact (0.480).

**Conclusion:**

The findings of this study suggested that nursing managers should develop an authentic leadership style, which can effectively improve nurses’ caring behaviors toward patients in clinical practice. Meanwhile, nursing leaders should strengthen nurses’ psychological evaluation and training, and promote nurses’ caring behavior in clinical settings.

## Background

The essence and cornerstone of the nursing profession is caring, which is one of the attributes necessary for providing care to patients [1]. Nursing scholars have explored theories of care for decades due to their significance to the nursing field [2, 3]. There are several concepts to define caring behavior, and one from Mosby’s Medical Dictionary is “actions characteristic of concern for the well-being of a patient, such as sensitivity, comforting, attentive listening, honesty, and non-judgmental acceptance” [4]. Conceptually, caring behavior comprises two main components. The first component is instrumental behavior, which is related to technical and physical behavior. The other component is expressive behavior, which comprises psychosocial and emotional behavior, including extending emotional kindness to patients and instilling loyalty, optimism, and confidence in them [5].

Previous studies indicate that caring behavior is an important indicator of quality nursing care and positive outcomes for patients and nurses. One study showed that patients’ perceptions of caring behavior and self-efficacy among those with cardiovascular disease were positively correlated. Furthermore, improving patients’ perceptions of nurses’ caring behavior could improve their self-efficacy and health [6]. A study reported that the higher the patients perceived nurses’ caring behavior, the higher their hospitalization satisfaction [7]. Another study revealed that nurses felt passionate about their confidence in developing caring relationships with patients that would enhance their job satisfaction [8]. Hospital administrators continuously endeavor to improve the quality of care due to the rapid advancement of technology and growing competition [9]. In hospitals, nurses make up the majority of healthcare professionals, and nurses’ caring behavior has a significant impact on the quality of patients’ care [10]. However, according to the relevant literature, neither the level of caring behavior perceived by patients nor the self-perceived caring behavior of nurses was high [5]. Therefore, to enhance the quality of care, nursing managers have been concentrating on how to promote nurses’ caring behavior. For this reason, identifying antecedents and mediators of caring behavior is crucial. Furthermore, it has been demonstrated that leadership style affects the attitudes and behaviors of employees [11].

## Authentic leadership and caring behavior

Authentic leadership upholds transparency, honesty, authenticity, and ethical standards when leading a team and interacting with followers. It serves as the cornerstone of all effective leadership styles and a contextual resource [12]. Walumbwa et al. proposed [13] that self-awareness, relational transparency, an internalized moral perspective, and balanced processing are the four elements of authentic leadership. Previous studies have shown that authentic leadership positively associated with employees’ creativity and work behaviors [14–16]. It is critical to foster authentic leadership in nurse managers to enhance nurses’ safety activities, reduce adverse patient outcomes, and promote care quality [17]. An integrative review shows that authentic leadership positively affects the organizational climate and relational social capital, promoting healthier work environments, greater organizational commitment, job satisfaction, and work engagement, and decreasing nurses’ absenteeism, mental exhaustion, burnout, and their intentions to quit jobs [18]. However, studies on the relationship between authentic leadership and caring behavior among Chinese nurses are limited.

### Authentic leadership and psychological capital

If authentic leadership affects nurses’ caring behavior, it essential to understand how it works and what the underlying mechanism is. We proposed a mediating model based on the Job Demand-Resource(JD-R) model. Bakker and Demerouti’s JD-R model defines job demands as those “physical, psychological, social, or organizational aspects of the job that require sustained physical and/or psychological (cognitive and emotional) effort or skills” [19]. Job resources are those aspects of the job that “are functional in achieving work goals; reduce job demands and the associated physiological and psychological costs; and stimulate personal growth”. Internal job resources (work autonomy, value, psychological capital, etc.) and external job resources (fringe benefits, leadership style, social support, etc.), are both helpful in promoting the individual development of positive work behavior. Based on the JD-R model, leaders’ leadership, as the provider of job resources, is a fundamental factor in determining employees’ work behavior [20]. Personal psychological capital is one form of an employee’s internal job resources [21], which would also be affected by leadership style and employees’ work behavior at the same time. Previous studies showed that “ authentic leadership is a significant antecedent variable of staff ’s psychological capital” [22–24].

## Psychological capital and caring behavior

In the context of positive psychology and organizational behavior, psychological capital was described as an individual’s positive psychological condition of development. It consists of self-efficacy, optimism, hope, and resiliency, which are the four fundamental psychological capacities [25]. It is widely regarded that personal psychological capital is a significant factor influencing how effectively a person performs at work [26]. According to previous studies, nurses with higher psychological capital would have better nursing performance outcomes [27–29]. Nursing performance outcomes refer to the extent to which nurses accomplish the objectives of healthcare organizations by acting in a professional capacity, and they may represent the actual performance of work related to all of the caring behavior-related activities [30]. However, there has been little research on whether psychological capital influences nurses’ caring behavior.

Based on literature reviews and the JD-R model, this study aimed to examine if psychological capital could act as a mediation between nursing managers’ authentic leadership and nurses’ caring behavior.

## Methods

### Design

This was a cross-sectional study that was reported following the STROBE Statement.

### Setting and participants

In December 2021, nurses recruited from 37 hospitals in Anhui Province participated in this study. The economic, social, and medical levels of Anhui Province are at the middle level in China. The 37 hospitals included 20 tertiary hospitals, 10 secondary hospitals, and 7 community hospitals. In China, tertiary hospitals are comprehensive and general hospitals with more than 500 beds at the city, provincial, or national levels. They deliver specialized health care, play a larger role in medical education and scientific research, and function as medical centers that provide care to multiple regions. The secondary hospitals are affiliated with a medium-sized city, county, or district with over 100 beds but less than 500. They are in charge of delivering comprehensive health care, medical education, and research on the local level. Community hospitals are typically township hospitals with under 100 beds and are responsible for delivering preventative care, primary health care, and rehabilitation services.

The inclusion criteria for participants were: (a) having worked as a registered nurse for longer than six months; (b) being presently employed in a hospital as a registered nurse; and (c) being willing to participate in the study. The exclusion criteria were as follows: nurses who were on sick leave, pregnant, or had physical or mental illnesses. The sample sizes were determined by considering the variables’ influencing factors and multiplying that number by ten [31]. In the survey, the general data included 11 items, and three questionnaires contained 60 items. Hence, we should collect 710 questionnaires from nurses and expand the sample size by 20% to offset the inadequate response. Therefore, the minimum sample size for this study should be at least 888.

### Measures

A four-part survey was utilized to collect data, including the general information questionnaire, the Authentic Leadership Questionnaire, the Psychological Capital Questionnaire, and the Caring Behaviour Inventory.

### General information questionnaire

It included 11 variables that reflected the nurses’ demographic and sociological characteristics, such as gender, age, years of employment, etc.

#### Authentic Leadership Questionnaire (ALQ)

The 16-item ALQ, created by Walumbwa [13] and translated by Lin [32], was used to investigate authentic leadership. Mao assessed the validity and reliability of the Chinese version of the ALQ [33]. The Chinese version of the ALQ includes four dimensions: self-awareness (four items), relational transparency (five items), morals and ethics (four items), and balanced processing (three items). In this questionnaire, answers are evaluated on a 5-point Likert scale from 1 (*strongly disagree*) to 5 (*strongly agree*). The higher the total score, the stronger the agreement. The Cronbach’s alpha coefficients for the ALQ in this survey were 0.96 overall, as well as 0.91, 0.90, 0.85, and 0.86 for each of the four dimensions.

#### Psychological Capital Questionnaire (PCQ)

The psychological capital of nurses was assessed using the Chinese version of the PCQ [25, 34]. The questionnaire comprises four dimensions: self-efficacy (six items), hope (six items), resilience (five items), and optimism (three items). Each item is scored from 1 (*strongly disagree*) to 6 (*strongly agree*). The total scores range from 20 to 120 points; the higher the score, the more psychological capital is represented. In this survey, Cronbach’s alpha coefficients for the PCQ and its four dimensions ranged from 0.72 to 0.92.

#### Caring Behavior Inventory (CBI)

The Chinese version of the CBI was utilized to evaluate nurses’ caring behavior [35]. The inventory comprises 24 items and four dimensions: (a) assurance, which is instantly available to respond to patients’ concerns and security; (b) knowledge and skill, which reveal diligence and competence; (c) respectfulness, which is related to the dignity of the individual; and (d) connectedness, which enables patients to receive support every time [35]. Each item is scored from 1 (*never*) to 6 (*always*). The total scores range from 24 to 144; the higher the score, the more caring the nurse was. Cronbach’s alpha coefficients for the CBI and its four dimensions in this study ranged from 0.89 to 0.96.

### Data collection

In December 2021, the Questionnaire Star online platform was used to make up online questionnaires and form a QR code for this survey. First, the project principal investigator trained 37 research assistants online from 37 hospitals. Training contents included the study purpose, importance, process evaluation, and noted points. Second, the survey QR code was then distributed to nurses via WeChat groups by 37 research assistants. The preface of the survey noted the study purpose, inclusion and exclusion criteria, informed consent, instructions, and contact information of the principal investigator. Participants would be excluded automatically if they filled out the inclusion criteria that did not match this study, and they could not enter the online survey again. A mobile phone number can only fill out one online survey, and all questions in the survey were set to be mandatory. Third, when a participant completed an online questionnaire, the principal investigator’s Questionnaire Star account would receive the data immediately. The QR code for this survey would automatically expire after seven days. Seven days later, the principal investigator would download all data from her Questionnaire Star account in Excel format.

### Statistical analysis

SPSS 20.0 and AMOS 21.0 were used for data analysis. The mean, standard deviation, frequency, and percentage were used to describe the scores of each variable and the basic characteristics of participants. The data were normally distributed in the study, wherein Pearson’s correlation coefficient (*r*) was used to conduct a correlation analysis of study variables.

Structural equation modeling (SEM) was used to confirm if psychological capital mediated the relationship between authentic leadership and the caring behavior of nurses. Based on the literature reviews and the JD-R model, caring behavior, authentic leadership, and psychological capital acted as the dependent variable, independent variable, and mediator in the SEM, respectively. According to Hu and Bentler’s recommendation [36], the following goodness-of-fit statistics were used to evaluate the fit degree of SEM and data: Root Mean Square Error of Approximation (*RMSEA*) with an acceptance level of<0.08, Comparative Fit Index (*CFI*) with an acceptance level of>0.90, Goodness of Fit Index (*GFI*) with an acceptance level of >0.90, Normal Fit Index (*NFI*) with an acceptance level of>0.90, Tucker-Lewis Index (*TLI*) with an acceptance level of>0.90 and Standardized Root Means Square Residuals (*SRMR*) with an acceptance level of < 0.05. Because the sample size was larger than 500, it is not considered the chi-square value (χ^2^) and χ^2^/df ratio.

A 5,000-sample bootstrapping procedure with a 95% confidence interval (*CI*) was used to estimate and test the mediating role [37]. The *p-value* was less than 0.05, which was considered significant. All significant tests were two-tailed with a 5% level of significance.

## Results

### Nurses’ basic characteristics and scores of authentic leadership, psychological capital, and caring behavior

We received 3,662 nurses’ surveys from 37 hospitals in Anhui Province. All collected surveys were checked by investigators, and 167 invalidated surveys were eliminated. In the end, 3,495 valid online surveys were collected, yielding a 95.44% valid response rate.

A descriptive analysis of nurses’ general demographic characteristics is shown in Table 1. The mean scores of authentic leadership, psychological capital, and caring behavior and their subdomain scores are shown in Table 2.

**Table 1:** Nurses’ basic characteristics (*n* = 3,495)

Variables	Categories	n	%
**Gender**			
	Women	3401	97.31
	Men	94	2.68
**Age(years)**			
	< 30	1184	33.87
	30～39	1612	46.12
	40～49	530	15.16
	≥ 50	169	4.83
**Years of working**			
	< 1	123	3.51
	1～2	152	4.34
	3～5	524	14.99
	6～10	921	26.35
	11～20	1162	33.24
	> 20	613	17.54
**Initial education level**			
	≤Associate degree	2596	74.27
	Bachelor degree	887	25.37
	≥ Master degree	12	0.34
**Marital status**			
	Married	2726	77.99
	Single	706	20.20
	Divorced or widowed	63	1.80
**Professional title**			
	Nurse	567	16.22
	Nurse practitioner	1369	39.17
	Nurse-in-charge	1362	38.96
	≥Associate professor of Nursing	197	5.63
**Departments**			
	Internal Medicine	835	23.89
	Surgery	837	23.94
	Obstetrics and Gynecology	286	8.18
	Pediatrics	159	4.54
	Emergency	180	5.15
	ICU	106	3.03
	Operating room	242	6.92
	Outpatient department	209	5.97
	Others	641	18.34
**Hospital level**			
	Tertiary	2006	57.39
	Secondary	1472	42.12
	Community hospital	17	0.48
**Nature of hospitals**			
	Public hospitals	3044	87.01
	Private hospitals	451	12.90
**Employment status**			
	Permanent	840	24.03
	Contract	1857	53.13
	Labour dispatch	798	22.83
**Monthly income (RMB)**			
	< 5000	1529	43.74
	5000～10000	1775	50.78
	> 10,000	191	5.46

**Table 2:** The total and subdomains scores of AL, PsyCap and CB

Variables and subdomains	M	SD
AL	52.04	13.24
Self-awareness	17.12	3.28
Relational transparency	21.11	4.40
Morals and ethics	17.09	3.29
Balanced processing	12.72	2.68
PsyCap	96.89	17.78
Self-efficacy	28.95	5.64
Hope	28.86	5.59
Resilience	24.39	4.53
Optimism	14.69	3.02
CB	104.28	17.01
Respectfulness	31.53	4.68
Connectedness	25.64	4.20
Knowledge and skill	27.19	3.53
Assurance	43.92	5.67

Notes: M = Mean, SD = Standard deviation, AL = authentic leadership, PsyCap = psychological capital, CB = caring behavior

### Correlation among authentic leadership, psychological capital, and caring behavior

Pearson’s correlation analysis showed that psychological capital, authentic leadership, and caring behavior were significantly positively correlated between the two ( *r* : 0.443 ~ 0.671, *p* < 0.01) (Table 3).

**Table 3:** Correlation among AL, PsyCap and CB

Variables	1	2	3
1. AL	1		
2. PsyCap	0.540^**^	1	
3. CB	0.433^**^	0.671^**^	1

Notes: ^**^p < 0.01, AL = authentic leadership, Psycap = psychological capital, CB = caring behavior

### SEM with a mediating role

**Fig. 1:**
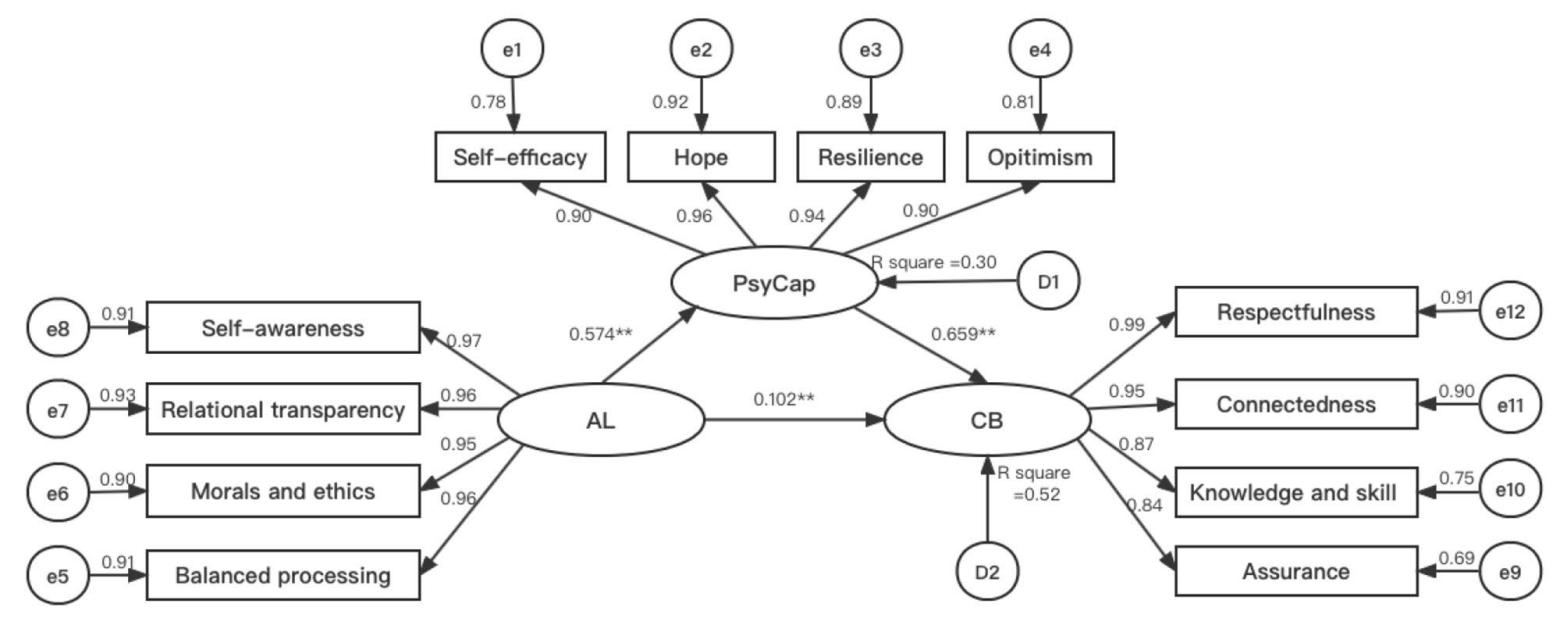
Structural equation modeling. Note: ^**^p < 0.001. AL = authentic leadership; PsyCap = psychological capital; CB = caring behavior

Figure 1 shows the SEM constructed for this study. This SEM included three latent variables (authentic leadership, psychological capital, and caring behavior) and 11 measured variables (self-awareness, relational transparency, morals and ethics, balanced processing, self-efficacy, hope, resilience, optimism, respectfulness, connectedness, knowledge and skill, and assurance). According to Hu and Bentler’s recommendation [36], the degree of fit between the SEM and the data was evaluated by goodness-of-fit statistics. In this model, *RMSEA* = 0.057, *CFI* = 0.991, *GFI* = 0.984, *NFI* = 0.990, *TFI* = 0.987, and *SRMR* = 0.030. The SEM fit the data acceptably.

First, without the inclusion of mediating variables (psychological capital), the overall impact of the independent variable (authentic leadership) on the dependent variable (caring behavior) was examined. The findings showed that authentic leadership perceived by nurses significantly influenced their caring behavior for patients (*β* = 0.102, *p <* 0.001). Second, the SEM demonstrated a statistically significant correlation between psychological capital and caring behavior (*β* = 0.659, *p* < 0.001), as well as authentic leadership (*β* = 0.574, *p <* 0.001).

We calculated a 95% *CI* of percentile bootstrapping through 5,000 bootstrap samples [37], which further verified the establishment of the mediation path of psychological capital between authentic leadership and caring behavior. It was found that the 95% *CI* of the indirect effect of authentic leadership on caring behavior was 0.350 ~ 0.402 (*p* < 0.001), so the mediation effect was established (Table 4). The path of authentic leadership and caring behavior was considerably and statistically significantly enhanced by 0.378 = 0.659*0.574 (*p* < 0.001) when a mediator variable (psychological capital) was included. This study revealed that authentic leadership was strongly associated with the caring behavior of nurses, with psychological capital functioning as a partial mediator between them. The psychological capital mediating role value was 0.378, accounting for 78.75% of the overall impact (0.480).

**Table 4:** Standardized direct, indirect, and total effect of the SEM.

Path	B	SE	95% CI	
			Lower	Upper
Standardized direct effect				
AL→CB	0.102	0.018	0.066	0.134
Standardized indirect effect				
AL→CB	0.378	0.013	0.350	0.402
Standardized total effect				
AL→CB	0.480	0.016	0.445	0.506

Notes: SEM = Structural equation modeling, AL = authentic leadership, CB = caring behaviour, SE = standard error, CI = confidence interval

## Discussion

The purpose of this study was to examine the mediating role of psychological capital on the relationship between authentic leadership and nurses’ caring behavior. The SEM confirmed that psychological capital partially mediated the relationship between authentic leadership and caring behavior (*β* = 0.378, *p* < 0.001, 95% *CI* = 0.350 ~ 0.402).

Nurses’ caring behavior could have a beneficial effect on patients, nurses, and organizations. Caring behavior proved critical in improving the quality of nursing care and enhancing patient safety. First, this survey showed that caring behavior among 3,495 nurses was at a moderate level; the result was lower than in a previous study in China [38]. The reasons may be related to the various areas investigated and the departments of nurses. The nurses in this study were recruited from Anhui, where the economic, social, and medical levels are at the middle level in China. Liu investigated the nurses’ caring behavior in the oncology departments of Shanghai and Nanjing [38]. The economic, social, and medical levels in Shanghai and Nanjing are significantly higher than those in Anhui Province.

Second, the results also indicated that authentic leadership had a significant and positive influence on the caring behavior of nurses. As the authentic leadership theory describes [14], authentic leaders create a trusted workplace by fairly evaluating all the relevant information before making decisions, allowing followers to participate in their own work, publicly disclosing information and their true thoughts and feelings, and encouraging others to do the same. They also take actions that are guided by their own internal ethical standards and values. The processes of self-awareness and self-regulation on the part of the leader then encourage the followers’ authenticity and improvement, resulting in the well-being and enhanced performance of the followers [39, 40]. This study added significantly to the knowledge because there was limited research on the correlation between caring behavior and authentic leadership in nursing.

Third, the results revealed a significant positive relationship between authentic leadership and nurses’ psychological capital. This outcome was in accordance with previous findings showing that authentic leadership by managers could enhance the psychological capital of the staff [22, 23, 41]. Authentic leaders often intentionally obey standards and policies, handle individual nurses with transparency, do not hide the truth, and treat all followers equitably. Meanwhile, authentic leaders support their staff’s self-improvement through guidance and support [13]. Consequently, nurses may strengthen their psychological resiliency, improve their self-efficacy, and get proactive, effective management support when faced with challenges. Thus, nurses are more optimistic and content about their situations and can keep positive attitudes for the future of hospitals, enhancing nurses’ psychological capital.

Fourth, this study supported the direct impact of psychological capital on nurses’ caring behavior, demonstrating that nurses who had high psychological capital performed in a more caring manner. According to the JD-R model, when employees believe there are actually enough resources to fulfill the demand for the work, performance and engagement improve [19]. As a part of employees’ positive psychological job resources, psychological capital enables workers to invest in their work actively and enthusiastically, sustaining their unwavering drive to accomplish work and exhibit work-related behaviors [21]. Based on previous findings, better performance may be achieved by nurses with better psychological capital. Since the processes that comprise caring behavior are related to nursing performance outcomes [30], this study provided evidence of the relationship between psychological capital and caring behavior among nurses.

Finally, the SEM indicated psychological capital played a partial mediating role in the relationship between authentic leadership and caring behavior. Therefore, in addition to being positively correlated with caring behavior, psychological capital also strengthened the influence of authentic leadership on nurses’ caring behavior. Nursing managers can activate nurses’ caring behavior by improving nurses’ psychological capital and the effectiveness of their authentic leadership. According to the JD-R model [19], job resources are classified as internal and external resources, both of which are helpful for encouraging self-improvement and positive behavior at work. Authentic leadership (an external resource) affects psychological capital (an internal resource), which promotes nurses’ caring behavior. Therefore, internal resources that are influenced by external factors affect an individual’s work behavior. As a result, the JD-R model is further enriched by these findings. Training and education in authentic leadership for nurse leaders and psychological capital for nurses could promote clinical nurses’ caring behavior for patients.

### Limitations

First, all data was collected using self-reported questionnaires, resulting in reporting bias. Nurses’ caring behavior should be evaluated from the objective perspectives of patients and nursing managers. Second, the influence of covariables was not considered in the SEM. The participants’ socio-economic and other contextual factors related to nurses (individual factors) and institutions (organizational culture) may mediate and affect the dependent variables of this study. In the future study, we should consider the covariables to design specific interventions for nurses. Third, we were unable to accurately pinpoint the causal relationship between the three variables since this study utilized a cross-sectional design. Longitudinal empirical studies are required to determine causal relationships and the differences between variables. Since this is a quantitative study, qualitative research is required to ensure an in-depth understanding of the influence of psychological capital and managers’ authentic leadership on nurses’ caring behavior.

## Conclusion

This study explored the relationship between authentic leadership, psychological capital, and caring behavior among Chinese nurses. The findings suggested that nursing managers should develop an authentic leadership style, which can effectively improve nurses’ caring behaviors toward patients in clinical practice. Meanwhile, nursing leaders should strengthen nurses’ psychological evaluation and training, further promoting nurses’ caring behavior.

## Data Availability

The datasets generated and/or analyzed during the current study are not publicly available due to the data consent with participants but are available from the corresponding author upon reasonable request.
